# Loneliness and type 2 diabetes incidence: findings from the English Longitudinal Study of Ageing

**DOI:** 10.1007/s00125-020-05258-6

**Published:** 2020-09-15

**Authors:** Ruth A. Hackett, Joanna L. Hudson, Joseph Chilcot

**Affiliations:** Health Psychology Section, Institute of Psychiatry, Psychology and Neuroscience, King’s College London, London, UK

**Keywords:** Loneliness, Prospective study, Social isolation, Type 2 diabetes

## Abstract

**Aims/hypothesis:**

Loneliness is associated with all-cause mortality and coronary heart disease. However, the prospective relationship between loneliness and type 2 diabetes onset is unclear.

**Methods:**

We conducted a longitudinal observational population study with data on 4112 diabetes-free participants (mean age 65.02 ± 9.05) from the English Longitudinal Study of Ageing. Loneliness was assessed in 2004–2005 using the revised University of California, Los Angeles (UCLA) Loneliness Scale. Incident type 2 diabetes cases were assessed from 2006 to 2017. Associations were modelled using Cox proportional hazards regression, adjusting for potential confounders, which included cardiometabolic comorbidities.

**Results:**

A total of 264 (6.42%) participants developed type 2 diabetes over the follow-up period. Loneliness was a significant predictor of incident type 2 diabetes (HR 1.46; 95% CI 1.15, 1.84; *p* = 0.002) independent of age, sex, ethnicity, wealth, smoking status, physical activity, alcohol consumption, BMI, HbA_1c_, hypertension and cardiovascular disease. Further analyses detected an association between loneliness and type 2 diabetes onset (HR 1.41; 95% CI 1.04, 1.90; *p* = 0.027), independent of depressive symptoms, living alone and social isolation. Living alone and social isolation were not significantly associated with type 2 diabetes onset.

**Conclusions/interpretation:**

Loneliness is a risk factor for type 2 diabetes. The mechanisms underlying this relationship remain to be elucidated.

**Figure:**
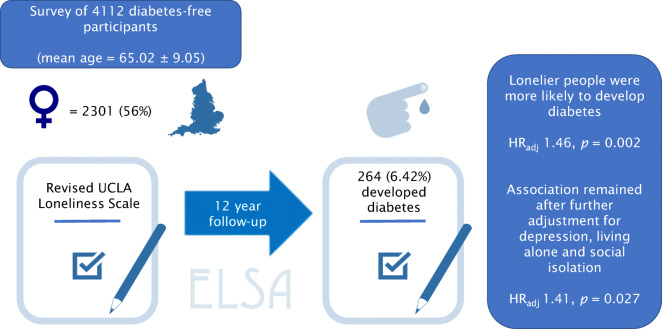
Graphical abstract

**Electronic supplementary material:**

The online version of this article (10.1007/s00125-020-05258-6) contains peer-reviewed but unedited supplementary material, which is available to authorised users.

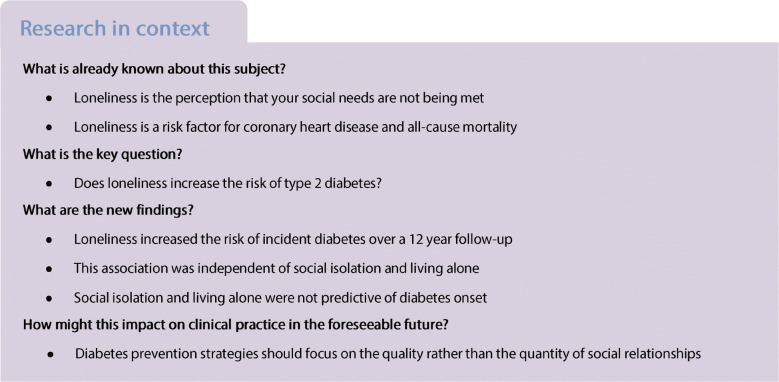



## Introduction

Loneliness is a negative emotion that occurs when an individual perceives that their social needs are not being met. It reflects an imbalance between desired and actual social relationships [1]. Survey data suggest that loneliness is a common experience, with a fifth of adults in the UK [2] and a third of adults in the USA [3] reporting feeling lonely sometimes.

There has been increasing research focused on loneliness as a determinant of health. Meta-analytic evidence suggests that loneliness is a predictor of all-cause mortality, indicating that lonely individuals have a 22% greater risk of death when compared with non-lonely individuals [4]. Loneliness has a negative effect on cardiovascular health and has been associated with incident CHD [5]. This is of relevance in type 2 diabetes, as CHD is a frequent complication of the condition and a leading cause of death in this population [6].

It is plausible that deleterious cardiometabolic factors associated with loneliness could contribute to type 2 diabetes [7]. Loneliness is associated with ageing [2, 3] and obesity [8], both of which are major risk factors for type 2 diabetes [9]. Further, evidence from large observational cohort studies indicates that loneliness is associated both cross-sectionally [8] and prospectively [10] with the metabolic syndrome. However, studies associating loneliness with HbA_1c_ have been less consistent [11, 12].

To date, no study has prospectively associated loneliness with incident type 2 diabetes, although there is evidence of a cross-sectional association [13, 14]. Some studies have investigated social isolation [15–19] or living alone [17, 20–22] as risk factors for type 2 diabetes. However, it is important to note that loneliness is not synonymous with social isolation as it relates to the perceived quality rather than quantity of social connections [1]. Further, there is evidence that loneliness and isolation are differentially associated with health outcomes [23, 24].

The majority of studies assessing social isolation [15, 16, 18, 19] and living alone [17, 21, 22] as risk factors for diabetes have failed to observe an association when taking potential confounding factors (such as health behaviours) into account. The German MONICA/KORA (MONitoring of Trends and Determinants in CArdiovascular Disease/Kooperative Gesundheitsforschung in der Region Augsburg (Cooperative Health Research in the Region of Augsburg) cohort of over 8000 participants found prospective associations between social isolation [18] and living alone [20] with incident diabetes, but only in male participants. A more recent analysis of this cohort found that poor social network satisfaction, a measure of relationship quality, increased the risk of type 2 diabetes in men only [25]. Interestingly, this association was independent of both social isolation and living alone.

The current study set out to address whether loneliness was a predictor of incident type 2 diabetes in a representative cohort of adults aged over 50 years living in England. We also aimed to assess whether social isolation and living alone were risk factors for type 2 diabetes. As the relationship between loneliness and social isolation is suggested to be weak to moderate for older people [24], we hypothesised that loneliness, social isolation and living alone would exert independent effects on type 2 diabetes risk.

Further, it is important to consider the impact of depression as a possible confounding variable in the relationship between loneliness and type 2 diabetes. Previous research indicates that loneliness has a reciprocal relationship with depression [26]. Depression is also a possible pathway through which loneliness impacts cardiometabolic health [10], with a large body of evidence suggesting that depressed individuals are more likely to develop type 2 diabetes than those without depression [27]. Given this, we considered depressive symptoms in our analyses.

## Methods

### Participants

The study used data from the English Longitudinal Study of Ageing (ELSA), a representative panel study of adults aged 50 and older living in England. Data collection began in 2002–2003 (wave 1), with follow-up waves biennially [28]. Self-reported questionnaire and interview data are collected at each wave and biological and anthropometric data are collected at alternate waves. Ethical approval for ELSA was obtained from the National Research Ethics Service. All participants provided informed consent.

**Fig. 1:**
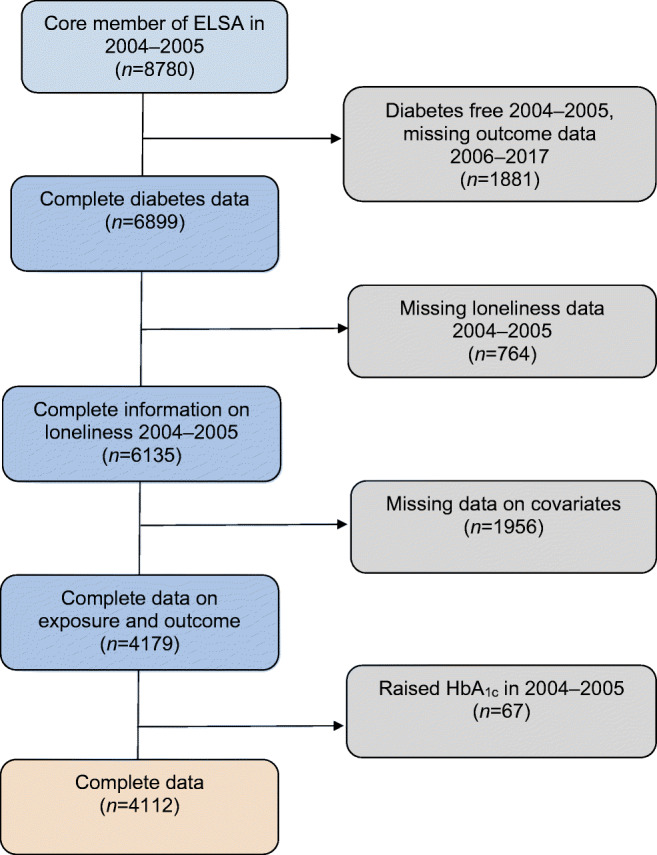
Flow diagram of participants included and excluded from the analyses

In the current study, we investigated the association between loneliness measured at wave 2 (2004–2005; the first wave in which loneliness was assessed) and incident type 2 diabetes from wave 3 (2006–2007) to wave 8 (2016–2017). Participants included in the analysis self-reported that they were free of diabetes/high blood sugar at baseline (2004–2005). The median follow-up time was 10 years. A total of 8780 participants took part in wave 2. Participants were included in our study if they had complete data on loneliness and covariates at baseline (2004–2005) and if they provided follow-up data on self-reported type 2 diabetes. Those with HbA_1c_ values in the diabetes range [9] (≥6.5%; 48 mmol/mol) at baseline were excluded. A flowchart of those included and excluded from the study can be found in Fig. 1. Our analytical sample was 4112 participants.

In comparison with those excluded from the analysis (*n* = 4668), those included were significantly less lonely, and were more likely to be younger, wealthier and of white ethnicity (*p* < 0.001). They were less likely to smoke, were more physically active and were less likely to have hypertension or CVD at baseline (*p* < 0.001). They had a lower BMI on average (*p* < 0.001) and were more likely to consume alcohol regularly than those excluded from the analysis (*p* = 0.002). No sex differences were evident (*p* = 0.098).

### Measures

#### Predictor variable: loneliness

We assessed loneliness with the three-item revised University of California, Los Angeles (UCLA) Loneliness Scale [29]. Participants rated items such as ‘*How often do you feel you lack companionship?*’ with response options of 1, ‘hardly ever/never’; 2, ‘some of the time’; and 3, ‘often’. Ratings were averaged to produce a score ranging from 1 to 3, with higher values indicating greater loneliness [23]. We also assessed loneliness as a continuous score (range 3–9) in supplementary analyses [8, 30]. The Cronbach’s α of the scale was 0.82 in our sample.

#### Outcome variable: type 2 diabetes incidence

Time to self-reported type 2 diabetes was assessed between wave 3 (2006–2007) and wave 8 (2016–2017). At each wave, participants were asked whether a physician had given them a diagnosis of diabetes or high blood sugar since their last interview. Time of diagnosis was indexed as the wave at which diabetes/high blood sugar was first reported. Time to event was measured in months from wave 2 (2004–2005) to the follow-up wave when diabetes/high blood sugar was mentioned. For those not diagnosed with diabetes by wave 8, time to censoring was the time from wave 2 to drop out.

#### Covariates

The covariates included in our analyses were measured at baseline (2004–2005). Participants self-reported their age, sex (man/woman) and ethnicity (white/non-white). We controlled for household non-pension wealth, which has been found to be the most relevant indicator of socioeconomic position for this cohort [28]. Wealth was divided into quintiles across the entire wave 2 sample. Participants self-reported whether they smoked (non-smoker/smoker), their frequency of physical activity (light or none weekly/moderate or vigorous once a week/moderate or vigorous more than once a week) and their alcohol consumption (≥5 times a week, <5 times a week). Height (cm) and weight (kg) were objectively measured during the nurse visit at wave 2 and used to calculate BMI (kg/m^2^). Participants self-reported whether they had received a doctor diagnosis of hypertension and this was combined with the objective nurse measure of blood pressure to create a binary variable (no/yes). We defined hypertension as systolic blood pressure ≥140 mmHg and diastolic blood pressure ≥90 mmHg. Participants self-reported whether they had angina, myocardial infarction or stroke, and we used this information to generate a measure of prevalent CVD (no/yes). HbA_1c_ was objectively measured during the nurse visit and samples were analysed at the Royal Victoria Infirmary laboratory, Newcastle upon Tyne, UK. HbA_1c_ values are reported in Diabetes Control and Complication Trial units (%) and International Federation of Clinical Chemistry units (mmol/mol).

### Secondary predictor variables

#### Depression

Depressive symptoms were measured using the eight-item Centre for Epidemiological Studies Depression Scale (CES-D) [31], where higher scores indicate greater symptoms. Items included statements such as ‘*I felt depressed*’ and ‘*My sleep was restless*’. We excluded the CES-D item on loneliness to avoid direct overlap with the loneliness scale. A dichotomous response to each item (0 = ‘no’; 1 = ‘yes’) resulted in a total score ranging from 0 to 7. In line with previous work [23], a score ≥6 was used to define severe depressive symptoms. We also assessed depressive symptoms as a total score in supplementary analyses [30]. The internal consistency of the measure was acceptable (α = 0.76).

#### Living alone and social isolation

Participants self-reported whether they lived alone (no/yes). Social isolation was measured using an index based on the extent of contact within a person’s social network and their involvement with social organisations [23, 30]. Participants were asked about frequency of contact with their children, other family and friends, with response options of ‘less than once a year/never’, ‘once or twice a year’, ‘every few months’, ‘once or twice a month’, ‘once or twice a week’ and ‘three or more times a week’. Participants received a point if they had less than monthly face-to-face or telephone contact with each of the three categories of social tie. Participants received another point if they did not participate in any social organisation (e.g. social or sports clubs, churches or residents’ groups). Total scores ranged from 0 to 4, with higher scores indicating greater isolation. Few participants received a score of 4, so we combined categories 3 and 4.

### Statistical analysis

Descriptive characteristics of the sample are presented as either mean (SD) or number (percentage). The characteristics of those who did and did not develop type 2 diabetes were compared using *t* tests for continuous variables and χ^2^ tests for categorical variables. Associations between loneliness and sample characteristics were assessed using Pearson’s correlations for continuous variables and univariate ANOVAs for categorical variables.

We established that the proportional hazards assumption was not violated using log (−log [survival]) vs log (time) graphs. Following this, we used Cox proportional hazards regression to investigate the association between loneliness and type 2 diabetes incidence, controlling for age, sex, wealth, ethnicity, smoking, physical activity, alcohol consumption, BMI, hypertension, CVD and HbA_1c_ (Model 1). Loneliness was inserted as a continuous variable where the HR and 95% CIs represent a 1 U increase.

In secondary analyses, additional covariates were added to the model to test the independent effect of loneliness on diabetes incidence. In Model 2, depression was added. In Model 3, living alone was included. In Model 4, social isolation was added. Model 5 was the final model and included loneliness, all covariates, depression, living alone and social isolation together as predictors of diabetes incidence. We conducted collinearity diagnostic tests to check for collinearity. Variable inflation factors were <1.26, suggesting collinearity was not present. For graphical purposes, total loneliness score (range 3–9) was dichotomised using a median split into low loneliness (scores of 3) and high loneliness (scores 4–9). Incident cases are plotted on a graph to reflect the time to diagnosis for these groups.

We conducted a sensitivity analysis to address the possibility of reverse causality by excluding participants who developed diabetes within 2 years of baseline (wave 3; 2006–2007). In supplementary analyses, we examined whether there was a moderating effect of age, sex or ethnicity on association between loneliness and type 2 diabetes by adding interaction terms to Model 1. Age was entered as a mean-centred interaction term. We also checked whether the pattern of results changed when entering loneliness and depression as continuous scores. Analyses were conducted using IBM SPSS Statistics for Macintosh, version 24 (IBM, Armonk, New York, USA).

## Results

### Participant characteristics

A total of 4112 participants took part in the study and, of these, 264 (6.42%) developed type 2 diabetes over the follow-up period. An overview of participant characteristics at baseline, along with a comparison of those who did and did not develop type 2 diabetes, can be found in Table 1. Those who developed diabetes were significantly lonelier (1.42 ± 0.53) on average than those who did not develop diabetes (1.33 ± 0.47; *p* = 0.013). They were more likely to be male (*p* = 0.001) and of non-white ethnicity (*p* = 0.018), and to be less well off financially (*p* = 0.001), than those who did not develop diabetes. Those in the diabetes group were significantly less likely to consume alcohol regularly (*p* = 0.025) and were more likely to have hypertension (*p* < 0.001) and CVD (*p* = 0.005) at baseline than those in the non-diabetes group. They also had a higher BMI (*p* < 0.001) and greater HbA_1c_ levels (*p* < 0.001) on average. Those who developed diabetes reported significantly higher depressive symptoms at baseline (1.57 ± 1.97) than those who did not develop diabetes (1.20 ± 1.61; *p* = 0.003). The groups did not differ in age, smoking, physical activity, social isolation or living alone at baseline (*p* > 0.073).

**Table 1:** Participant characteristics (2004–2005) according to diabetes status (2006–2017)

Characteristic	Overall (*N* = 4112)	No diabetes (*n* = 3848)	Diabetes case (*n* = 264)	*p* value
Loneliness score	1.34 ± 0.48	1.33 ± 0.47	1.42 ± 0.53	0.013
Age (years)	65.02 ± 9.05	65.05 ± 9.09	64.62 ± 8.46	0.450
Sex (% women)	2301 (56)	2180 (56.7)	121 (45.8)	0.001
Wealth (£)	3.31 ± 1.38	3.33 ± 1.38	3.03 ± 1.38	0.001
Quintile 1	591 (14.4)	536 (13.9)	55 (20.9)	
Quintile 2	639 (15.5)	603 (15.7)	36 (13.6)	
Quintile 3	851 (20.7)	787 (20.5)	64 (24.2)	
Quintile 4	977 (23.8)	913 (23.7)	64 (24.2)	
Quintile 5	1054 (25.6)	1009 (26.2)	45 (17.1)	
Ethnicity (% white)	4074 (99.1)	3816 (99.2)	258 (97.7)	0.018
Smoker (% yes)	550 (13.4)	508 (13.2)	42 (15.9)	0.211
Physical activity				0.073
Light/none weekly	653 (15.88)	598 (15.6)	55 (20.8)	
Moderate	1002 (24.37)	940 (24.4)	62 (23.5)	
Vigorous	2457 (59.75)	2310 (60)	147 (55.7)	
Alcohol (% ≥5 days/week)	1033 (25.1)	982 (25.5)	51 (19.3)	0.025
BMI (kg/m^2^)	27.51 ± 4.61	27.31 ± 4.50	30.50 ± 5.08	<0.001
Hypertension (% yes)	1687 (41)	1536 (39.9)	151 (57.2)	<0.001
CVD (% yes)	481 (11.7)	436 (11.3)	45 (17)	0.005
HbA_1c_				<0.001
mmol/mol	35.80 ± 3.60	35.6 ± 3.60	39.1 ± 3.80	
%	5.43 ± 0.33	5.41 ± 0.33	5.73 ± 0.35	
Depression score^a^	1.23 ± 1.64	1.20 ± 1.61	1.57 ± 1.97	0.003
Depression case (% yes)^a^	162 (3.9)	142 (3.7)	20 (7.6)	0.002
Living alone (% yes)	909 (22.1)	855 (22.2)	54 (20.5)	0.504
Social isolation index^b^				0.556
0 (%)	383 (11.8)	362 (12)	21 (10)	
1 (%)	1616 (49.9)	1513 (50)	103 (49)	
2 (%)	947 (29.2)	885 (29.2)	62 (29.5)	
3 (%)	293 (9.1)	269 (8.8)	24 (11.5)	

Data are presented as mean ± SD or *n* (%)

^a^*n* = 4104

^b^*n* = 3239

We investigated associations between loneliness and demographic and clinical characteristics. Loneliness was significantly positively associated with age (*r* = 0.05, *p* < 0.001), HbA_1c_ (*r* = 0.03, *p* = 0.027) and depressive symptoms (*r* = 0.45, *p* < 0.001). Lonelier participants were more likely to be female (*F* [1,1440] = 6.58; *p* = 0.010) and non-white (*F* [1,1440] = 46.01; *p* < 0.001) than less lonely participants. Loneliness was associated with a greater likelihood of smoking (*F* [1,1440] = 15.28; *p* < 0.001) and physical inactivity (*F* [2,1409] = 22.51; *p* < 0.001), as well as a reduced likelihood of regular alcohol consumption (*F* [1,1440] = 16.28; *p* < 0.001). Lonelier participants were also more likely to have CVD (*F* [1,1440] = 22.91; *p* < 0.001) and to live alone (*F* [1,1440] = 364.93; *p* < 0.001) than less lonely participants. No significant associations between loneliness and BMI, hypertension or social isolation were observed.

### Loneliness (2004–2005) and type 2 diabetes incidence (2006–2017)

**Fig. 2:**
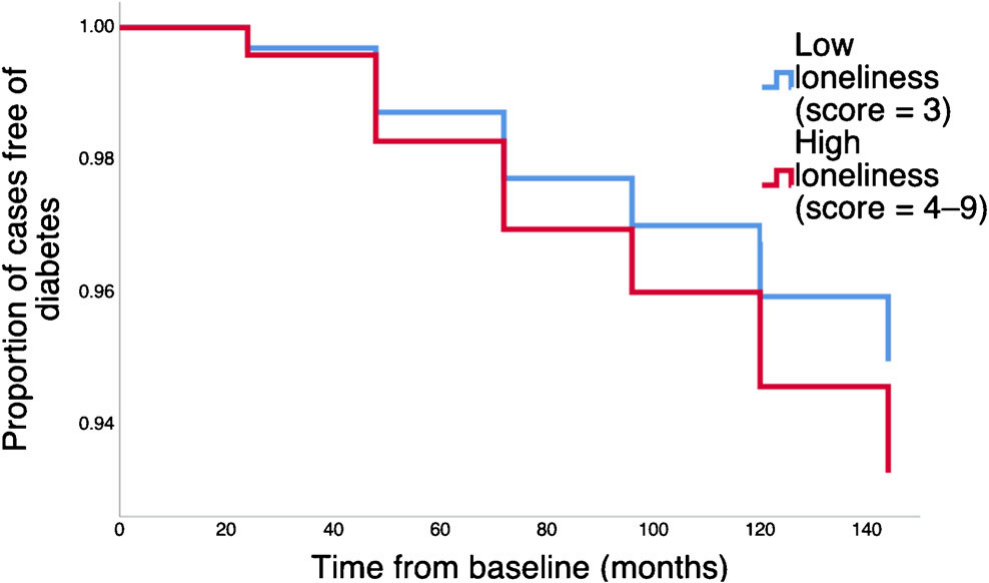
Survival curve of loneliness on type 2 diabetes incidence

The findings from the Cox regression models can be found in Table 2. Loneliness was a significant predictor of incident type 2 diabetes over the follow-up period (HR 1.46; 95% CI 1.15, 1.84; *p* = 0.002) independent of age, sex, ethnicity, wealth, smoking, physical activity, alcohol consumption, BMI, HbA_1c_, hypertension and CVD (Model 1). As can be seen in Model 2, the association between loneliness and later type 2 diabetes was robust to adjustment for depressive symptoms (HR 1.42; 95% CI 1.10, 1.84; *p* = 0.008). Living alone (Model 3) and social isolation (Model 4) were not significant predictors of type 2 diabetes. Our final model (Model 5) shows the independent association between loneliness and type 2 diabetes onset (HR 1.41; 95% CI 1.04, 1.90; *p* = 0.027), controlling for a range of covariates, as well as depressive symptoms, living alone and social isolation. A one point increase in the averaged loneliness score was associated with a 41% increase in the hazard of type 2 diabetes onset (95% CI estimate between 4% and 90%). A graphical representation of the Model 5 findings can be found in Fig. 2. The associations did not vary by age, sex or ethnicity (see electronic supplementary material [ESM] Table 1).

**Table 2:** Cox proportional hazards regression of loneliness, living alone and social isolation (2004–2005) on diabetes incidence (2006–2017)

Variable	Model 1 (HR; 95% CI) (*n* = 4112)	Model 2 (HR; 95% CI) (*n* = 4104)	Model 3 (HR; 95% CI) (*n* = 4112)	Model 4 (HR; 95% CI) (*n* = 3239)	Model 5 (HR; 95% CI) (*n* = 3233)
Loneliness	1.46 (1.15, 1.84)**	1.42 (1.10, 1.84)**	1.51 (1.18, 1.93)***	1.41 (1.08, 1.84)*	1.41 (1.04, 1.90)*
Age	0.99 (0.98, 1.02)	0.99 (0.98, 1.01)	0.99 (0.98, 1.01)	0.99 (0.97, 1.01)	0.99 (0.97, 1.01)
Sex (ref: men)	0.51 (0.40, 0.66)***	0.51 (0.39, 0.66)***	0.52 (0.40, 0.67)***	0.50 (0.37, 0.66)***	0.50 (0.37, 0.66)***
Wealth (ref: quintile 1)	1	1	1	1	1
2	0.73 (0.48, 1.13)	0.73 (0.47, 1.12)	0.74 (0.48, 1.13)	0.76 (0.47, 1.22)	0.75 (0.47, 1.21)
3	0.88 (0.61, 1.28)	0.87 (0.60, 1.26)	0.88 (0.61, 1.27)	0.91 (0.61, 1.37)	0.89 (0.59, 1.34)
4	0.85 (0.58, 1.24)	0.85 (0.58, 1.24)	0.84 (0.58, 1.23)	0.75 (0.48, 1.15)	0.74 (0.48, 1.15)
5	0.53 (0.35, 0.80)**	0.52 (0.34, 0.79)**	0.52 (0.34, 0.79)**	0.48 (0.30, 0.77)**	0.47 (0.30, 0.76)**
Ethnicity (ref: white)	2.69 (1.19, 6.09)*	2.69 (1.18, 6.10)*	2.71 (1.20, 6.14)*	2.16 (0.79, 5.87)	2.17 (0.80, 5.90)
Smoking (ref: non-smoker)	0.88 (0.62, 1.25)	0.90 (0.63, 1.27)	0.89 (0.63, 1.26)	0.81 (0.55, 1.21)	0.83 (0.56, 1.22)
Physical activity (ref: light/none)	1	1	1	1	1
Moderate	0.84 (0.58, 1.23)	0.87 (0.59, 1.27)	0.84 (0.58, 1.23)	0.72 (0.47, 1.10)	0.74 (0.48, 1.13)
Vigorous	0.87 (0.63, 1.22)	0.90 (0.64, 1.25)	0.87 (0.63, 1.21)	0.76 (0.53, 1.09)	0.78 (0.54, 1.13)
Alcohol (ref ≥5 days/week)	0.95 (0.69, 1.31)	0.95 (0.70, 1.31)	0.95 (0.69, 1.31)	0.90 (0.62, 1.29)	0.90 (0.63, 1.30)
BMI	1.09 (1.06, 1.12)***	1.09 (1.06, 1.11)***	1.09 (1.06, 1.12)***	1.09 (1.06, 1.12)***	1.08 (1.05, 1.11)***
Hypertension case (ref: no)	1.35 (1.04, 1.74)*	1.36 (1.05, 1.76)*	1.35 (1.05, 1.75)*	1.27 (0.95, 1.70)	1.28 (0.96, 1.72)
CVD case (ref: no)	1.01 (0.72, 1.41)	0.99 (0.71, 1.40)	0.99 (0.70, 1.39)	1.15 (0.80, 1.66)	1.14 (0.79, 1.65)
HbA_1c_	14.40 (9.74, 21.26)***	14.42 (9.75, 21.33)***	14.37 (9.73, 21.23)***	12.99 (8.37, 20.15)***	13.01 (8.36, 20.23)***
Depression (ref: no)		1.18 (0.79, 1.96)			1.12 (0.63, 1.97)
Living alone (ref: no)			0.85 (0.61, 1.18)		0.94 (0.65, 1.35)
Social isolation index (ref: 0)				1	1
1				1.06 (0.66, 1.71)	1.05 (0.65, 1.70)
2				1.24 (0.75, 2.06)	1.23 (0.74, 2.04)
3				1.53 (0.85, 2.76)	1.52 (0.84, 2.74)

*p < 0.05

**p < 0.01

***p < 0.001

Ref, reference category

### Sensitivity analysis: loneliness (2004–2005) and type 2 diabetes incidence (2006–2017)

We conducted a sensitivity analysis excluding participants who reported type 2 diabetes diagnosis within 24 months of the baseline assessment. As can be seen in Table 3, loneliness remained a significant predictor of incident diabetes (HR 1.54; 95% CI 1.11, 2.13; *p* = 0.009) independent of covariates, depressive symptoms, living alone and social isolation. We also assessed whether entering loneliness and depressive symptoms as continuous scores altered the results. The findings remained consistent when treating the measures in this way (see ESM Table 2).

**Table 3:** Sensitivity analysis showing Cox proportional hazards regression of loneliness, living alone and social isolation (2004–2005) on diabetes incidence (2008–2017)

Variable	HR (95% CI) (*n* = 3233)
Loneliness	1.54 (1.11, 2.13)**
Age	0.99 (0.97, 1.01)
Sex (ref: men)	0.50 (0.36, 0.68)***
Wealth (ref: quintile 1)	1
2	0.65 (0.37, 1.12)
3	1.03 (0.67, 1.59)
4	0.78 (0.49, 1.23)
5	0.48 (0.29, 0.80)**
Ethnicity (ref: white)	2.42 (0.88, 6.63)
Smoking (ref: non-smoker)	0.81 (0.52, 1.24)
Physical activity (ref: light/none)	1
Moderate	0.75 (0.47, 1.19)
Vigorous	0.82 (0.55, 1.21)
Alcohol (ref ≥5 days/week)	0.76 (0.50, 1.15)
BMI	1.09 (1.06, 1.13)***
Hypertension case (ref: no)	1.28 (0.93, 1.75)
CVD case (ref: no)	1.10 (0.73, 1.65)
HbA_1c_	12.30 (7.59, 19.91)***
Depression (ref: no)	1.12 (0.61, 2.06)
Living alone (ref: no)	0.98 (0.66, 1.45)
Social isolation index (ref: 0)	1
1	1.02 (0.61, 1.68)
2	1.10 (0.64, 1.88)
3	1.49 (0.79, 2.80)

**p < 0.01

***p < 0.001

Ref, reference category

## Discussion

To our knowledge, this study is the first to examine the association of loneliness with later type 2 diabetes incidence. Our findings show that loneliness is a robust predictor of type 2 diabetes incidence over 12 years of follow-up independent of a range of covariates, including sociodemographic factors, health behaviours and cardiometabolic comorbidities. This association was upheld when depressive symptoms were taken into account. We also assessed loneliness, social isolation and living alone simultaneously as predictors of type 2 diabetes incidence. In this analysis, loneliness remained an independent predictor of later type 2 diabetes. No significant associations for social isolation or living alone were observed.

No previous study has prospectively associated loneliness with incident type 2 diabetes, although this relationship has been assessed cross-sectionally [13, 14]. One analysis of 8593 older people living in Denmark found that loneliness was associated with diabetes in women only [13]. However, a larger cohort of over 20,000 Swiss nationals observed an association between loneliness and diabetes in both male and female participants [14]. Cross-sectional analyses cannot determine whether loneliness stimulates type 2 diabetes onset, or whether loneliness is an emotional manifestation of the strain of diabetes diagnosis on close social relationships. Our prospective results therefore add to the literature in establishing that loneliness is a predictor of type 2 diabetes incidence, independent of baseline HbA_1c_. Given the observational nature of this study, causality cannot be inferred. Our sensitivity analysis excluding cases of type 2 diabetes reported within 2 years of baseline aimed to address the risk of reverse causality. The observation that the association between loneliness and incident type 2 diabetes remained after these more immediate cases were excluded adds weight to the temporal sequence.

Social isolation or living alone were not independent risk factors for type 2 diabetes onset in this study. This result is in keeping with the majority of previous studies, which have also failed to observe an association between social isolation [15, 16, 18, 19] or living alone [17, 21, 22] and type 2 diabetes incidence when taking sociodemographic factors, health behaviours and clinical characteristics into account. Our findings are in contrast with analyses from the MONICA/KORA Augsburg cohort, where prospective associations of social isolation [18] and living alone [20] with incident diabetes were observed in male participants. We did not observe a moderating effect of sex on the relationship between social isolation or living alone and diabetes incidence (data not shown). There were some differences in the measure of social isolation employed in the studies, which may have contributed to the diverging results. Both measures included frequency of contact with social ties and organisation membership. However, our index was unweighted and did not include living alone as we preferred to assess the predictive value of this factor independently.

There is better concordance between the findings of the present study and a more recent analysis of the MONICA/KORA Augsburg cohort [25]. This study assessed perceived relationship quality by asking 6839 participants to rate their satisfaction with friends and relatives on a one item scale. Over 14 years of follow-up, men with lower social network satisfaction had a greater risk of type 2 diabetes than those with higher satisfaction ratings. This association was robust to adjustment for social isolation and living alone. Similarly, in the current study we found that loneliness was a predictor of incident type 2 diabetes, independent of social isolation or living alone. This finding highlights the need to examine loneliness, social isolation and living alone as distinct risk factors for poor health outcomes [23, 24]. It also supports previous work suggesting that these factors may be only weakly related for older adults [24].

Depression is the mostly widely studied psychosocial risk factor for diabetes [7], and loneliness and depression are suggested to have a reciprocal relationship [26]. Therefore, we considered depressive symptoms as a potential confounder of the relationship between loneliness and type 2 diabetes risk. Our findings suggest that loneliness increases the risk of type 2 diabetes, independently of depressive symptomology. This is in keeping with the idea that loneliness and depression are distinct constructs [26].

The mechanisms through which loneliness serves to increase the risk of type 2 diabetes remain to be elucidated. Theoretical work in this area suggests that loneliness is characterised by maladaptive hypervigilance for social threats [1]. This cognitive bias leads lonely individuals to perceive the social world as threatening, leading to patterns of inappropriate social behaviour that may evoke negative responses from peers, which reinforce the bias. Poor health behaviours are suggested to be one pathway through which the maladaptive hypervigilance of loneliness can impact health [1]. In a previous analysis of the ELSA cohort, loneliness was associated with an increased likelihood of smoking and physical inactivity [30], as well as obesity [8]. However, most studies that have associated loneliness with ill health have taken these factors into account in their analyses [4, 5]. Our findings were independent of smoking, physical inactivity, alcohol consumption and BMI.

Another possibility is that direct biological mechanisms may be involved in associating loneliness with ill health [1]. Frequent activation of stress-related biological systems as a result of chronic loneliness could lead to ‘wear and tear’ on the body resulting in dysregulation across multiple biological systems. For example, loneliness has been associated with disturbances in cortisol in naturalistic [32] and experimental settings [33] in healthy samples. Cortisol plays an important mechanistic function related to type 2 diabetes [7] and dysregulation in daily cortisol output is predictive of new onset pre-diabetes and type 2 diabetes [34]. In samples with overt type 2 diabetes, loneliness has been associated with dysregulation in cortisol responses to acute laboratory stress [35]. Loneliness is also associated with inflammation [36], which is of relevance to type 2 diabetes as pooled evidence suggests that heightened inflammation is a risk factor for the condition [37]. Indeed, loneliness has been associated with heightened inflammation in laboratory settings in people with diagnosed type 2 diabetes [35].

Our findings must be considered in terms of strengths and weaknesses. Our sample was drawn from a longitudinal nationally representative cohort which allowed the examination of type 2 diabetes incidence over a relatively long follow-up period. The analyses took a variety of potential confounding variables into account and we included several measures of social integration to assess the impact of the quality and the quantity of social connections on type 2 diabetes risk.

However, our study was not without limitations. Our data were observational and therefore we cannot infer causality. The strength of the association was small. Our measures were brief and likely do not capture the full complexity of experiences of loneliness, social isolation or living alone. Further, loneliness was only assessed at one timepoint, meaning our measure could reflect transient rather than persistent loneliness. However, theoretical work in the field suggests loneliness is relatively stable over time and may reflect a dispositional tendency [1]. Depression was not associated with diabetes incidence in our study, as previously reported in this cohort [38]. It is possible that our loneliness measure better reflects the English cultural expression of low mood than the depression measure used in this cohort. Our measure of type 2 diabetes was based on self-report rather than objective records; however, previous work suggests there is a high concordance between self-reported and clinically derived diabetes diagnoses [39]. The precise timing of diabetes onset was unknown. The assumption that interval survival times are exact can lead to biased estimates. Missing data are unavoidable in general population cohorts such as ELSA. We excluded participants with missing data. Those who were included were healthier, wealthier and less lonely than those who had dropped out, meaning selection bias due to non-random exclusion is possible. This may limit the generalisability of our findings. Finally, as there are few ethnic minority participants in ELSA, our findings may not generalise to non-white populations.

The current study highlights loneliness as a risk factor for type 2 diabetes for the first time. Further work is required to understand the potential causal nature of this relationship, as well as underlying mechanisms. There has been increasing interest in designing interventions to alleviate loneliness, with the most promising results detected for studies addressing maladaptive social cognitions, particularly through the use of cognitive behavioural therapy [40]. In line with our results, prevention strategies should focus on the quality rather than the quantity of social relationships, as increasing social contact is unlikely to alleviate feelings of loneliness [40]. It remains to be discovered whether these types of interventions or policies to address loneliness in older people could help prevent the onset of type 2 diabetes.

## Electronic supplementary material

ESM Tables (PDF 170 kb)

## Data Availability

Data from the English Longitudinal Study of Ageing are freely available to download from the UK Data Service at https://ukdataservice.ac.uk/.

## Abbreviations

CES-D: Centre for Epidemiological Studies Depression Scale
ELSA: English Longitudinal Study of Ageing
MONICA/KORA: MONitoring of Trends and Determinants in CArdiovascular Disease/Kooperative Gesundheitsforschung in der Region Augsburg (Cooperative Health Research in the Region of Augsburg)
