# Supplementary material for: Loneliness and type 2 diabetes incidence: findings from the English Longitudinal Study of Ageing
Source: Diabetologia. 2020 Sep 15;63(11):2329–38. doi: 10.1007/s00125-020-05258-6 (PMC7527325; doi:10.1007/s00125-020-05258-6)
Supplement: Supplementary file 1 — (PDF 170 kb) [file 125_2020_5258_MOESM1_ESM.pdf]

ESM Table 1: Cox proportional hazards regression of loneliness (2004–05) on diabetes incidence (2006–17) with interaction terms

	HR; 95% CI (n = 4112)
**Loneliness**	1.45;1.02-2.07*
**Age**	1.02;0.98-1.06
**Sex (ref: men)**	0.54;0.26-1.10
**Wealth (ref: quintile 1)**	1
**2**	0.78;0.51-1.21
**3**	0.93;0.64-1.35
**4**	0.81;0.55-1.20
**5**	0.54;0.35-0.82**
**Ethnicity (ref: white)**	1.57;0.15-16.55
**Smoking (ref: non-smoker)**	0.94;0.66-1.33
**Physical activity (ref: light/none)**	1
**Moderate**	0.81;0.55-1.18
**Vigorous**	0.87;0.62-1.22
**Alcohol (ref ≥ 5 days/week)**	0.94;0.68-1.30
**BMI**	1.09;1.06-1.12***
**Hypertension case (ref: no)**	1.39;1.07-1.80*
**CVD case (ref: no)**	1.03;0.73-1.45
**HbA_{1c}**	14.46;9.74-21.47***
**Loneliness*age**	0.98;0.96-1.01
**Loneliness*sex**	0.97;0.60-1.55
**Loneliness*ethnicity**	1.40;0.37-5.36

BMI= Body mass index; CI= Confidence Interval; CVD= Cardiovascular disease; **HbA_{1c}** = Glycated haemoglobin; HR= Hazard Ratio; Ref = reference category
*$p < 0.05$ **$p < 0.01$ ***$p < 0.001$

ESM Table 2: Cox proportional hazards regression of loneliness (2004–05) on diabetes incidence (2006–17) with loneliness and depression entered as continuous scores

	**HR; 95% CI** **(*n* = 4104)**
**Loneliness (range 3-9)**	1.11;1.01-1.21*
**Age**	1.00;0.98-1.01
**Sex (ref: men)**	0.50;0.39-0.65***
**Wealth (ref: quintile 1)**	1
**2**	0.73;0.47-1.13
**3**	0.87;0.60-1.26
**4**	0.86;0.60-1.26
**5**	0.53;0.35-0.80**
**Ethnicity (ref: white)**	2.63;1.16-5.97*
**Smoking (ref: non-smoker)**	0.90;0.64-1.28
**Physical activity (ref: light/none)**	1
**Moderate**	0.88;0.60-1.28
**Vigorous**	0.91;0.65-1.27
**Alcohol (ref ≥ 5 days/week)**	0.96;0.70-1.32
**BMI**	1.09;1.06-1.11***
**Hypertension case (ref: no)**	1.36;1.05-1.75*
**CVD case (ref: no)**	0.98;0.70-1.38
**HbA_{1c}**	14.41;9.74-21.33***
**Depression (range 0-7)**	1.05; 0.97-1.13

BMI= Body mass index; CI= Confidence Interval; CVD= Cardiovascular disease; **HbA_{1c}** = Glycated haemoglobin; HR= Hazard Ratio; Ref = reference category
*p < 0.05 **p < 0.01 ***p < 0.001
